# Evaluating the Effect of Forage Rape (*Brassica napus*) Ensiling Kinetics on Degradability and Milk Performance as Non-conventional Forage for Dairy Buffalo

**DOI:** 10.3389/fvets.2022.926906

**Published:** 2022-06-28

**Authors:** Mohamed Abdelrahman, Wei Wang, HaiMiao Lv, Zhou Di, Zhigao An, Wang Lijun, Aftab Shaukat, Wang Bo, Zhou Guangsheng, Yang Liguo, Hua Guohua

**Affiliations:** ^1^Key Lab of Agricultural Animal Genetics, Breeding and Reproduction of Ministry of Education, Huazhong Agricultural University, Wuhan, China; ^2^Animal Production Department, Faculty of Agriculture, Assuit University, Asyut, Egypt; ^3^College of Plant Science and Technology, Huazhong Agricultural University, Wuhan, China; ^4^National Center for International Research on Animal Genetics, Breeding and Reproduction (NCIRAGBR), Key Laboratory of Smart Farming for Agricultural Animals, Huazhong Agricultural University, Wuhan, China; ^5^Hubei Province's Engineering Research Center in Buffalo Breeding and Products, Wuhan, China

**Keywords:** silage, ruminal degradability, buffalo (*Bubalus bubalis*), milk performance, *Brassica napus*

## Abstract

The recent increase in demand for animal protein sources has led to the urgency to introduce non-conventional feed sources and opened the space to study feed management and its effects on animal productivity. Forage rape (*Brassica napus* L.) is a high-quality forage crop with a remarkable nutritional value and productive and fast growth capacity; however, studies on processing methods are limited. This study evaluates the effect of an ensiling process on rape silage quality kinetics, *in situ* degradability, and milk responses in dairy buffaloes. Firstly, the whole-plant forage rape was ensiled, and silage samples were collected 30, 60, and 90 days after ensiling to determine pH, evaluation of sensory characteristics, and chemical composition. Then, samples were taken for further chemical analysis at days 30, 60, and 90. After that, the degradability of the dry matter (DM) and crude protein (CP) of the silage was evaluated by an *in situ* degradability experiment using three fistulated buffalos (550 ± 20 kg body weight, 4.7 ± 0.76 years). Finally, whole-plant rape silage (after 60 days) was included in a 10, 20, and 30% of DM dairy buffalo diet in the lactating buffalo ration. The results showed that silage pH did not change significantly during the ensiling process (*p* > 0.05); however, the silage achieved the optimal comprehensive sensory characteristic score from days 30 to 60. There was also a significant change in neutral detergent fiber (NDF) content and acid detergent fiber content, which decreased significantly (*p* = 0.001 and *p* < 0.001, respectively). Ensiling of the whole-plant rape significantly reduced effective DM degradability (*p* < 0.05) without altering CP degradability (*p* > 0.05). Furthermore, the inclusion of forage rape silage linearly (*p* = 0.03) increased milk fat and protein contents and did not affect milk yield, lactose, and urea nitrogen contents in raw buffalo milk. In conclusion, whole-plant rape silage could significantly maintain the optimal ether extract (EE) protein content without affecting CP degradability, in addition to improving milk fat and milk protein. Therefore, ensiling may be an efficient method of forage rape utilization, and forage rape silage can be recommended as a good forage source for dairy buffaloes.

## Introduction

Feed resource limitations are becoming the main obstacle for the livestock production sector, which threatens future expansion opportunities due to increasing global demand for meat and milk. Therefore, the introduction of new forage resources can play an essential role in feed safety and support food production sustainability. For example, forage rape (*Brassica napus*) is a high-quality green forage capable of providing fast-growing and plentiful feed (1, 2). According to the European Economic Community (EEC), the global rapeseed yield reached 68 million tons in 2021/2022, where Canada ranked first globally in terms of total rapeseed yield (28.04%), followed by the EEC (25.10%), China (19.34%), India (11.37%), and Ukraine (4.87%) (3). Also, forage rape is known as “double-low” rape (4, 5) because, compared to traditional rape, forage rape has a lower content of erucic acid (<3%) and glucosinolate (<30 μmol/g) (6). These two compounds are the main anti-nutritive factors found in rape, which reduce feed acceptability and cause goiter, metabolic disorders, and even death. Moreover, forage rape can be intercropped in a pasture-based system due to its higher feed quality during a decline in the yield of other pasture crops such as ryegrass, in addition to high nutritional characteristics, i.e., higher metabolizable energy (ME), crude protein (CP), lower neutral detergent fiber (NDF) content, and reduced CH_4_ production (7). In addition, the relatively low cost of producing forage rape promoted this crop as an economical choice for fast-growing feed in resource-limited regions.

There are two significant ways to use rape as feed, the first is to use rapeseed as a meal. Rapeseed meal or canola meal (CM) is now widely used in North American dairy rations as a cost-effective alternative to soybean meal (SBM) (8). Compared to SBM and other protein sources, the inclusion of CM has been shown to increase nitrogen (N) utilization and production performance when fed to lactating dairy cows (9). The second form of rapeseed used as feed is rape straw as a roughage source. As CP content is an essential criterion for evaluating feed quality; rape straw contains (5.24%) CP, which is relatively higher than corn stalks (5%) and wheat straw (3.6%), and also higher than the average of roughage feeds used for ruminants (2.57%) (10).

Nevertheless, the proportion of rape stalks used as fodder feed is relatively low (<10%). Also, rape stalks are burned or wasted in the field, which causes many environmental pollutions, such as smoke. With the exception of rape straw, no previous reports have discussed the processing of whole-plant rape and its effect on improving silage quality and avoiding misuse of by-products.

Although previous works studied the effect of including forage rape silage in buffalo diets as a replacement for other silage sources (11) and did not discuss the dynamics during the ensiling process. Such changes during the ensiling process will alter the chemical analysis and, in turn, the feed properties, which will reflect animal performance.

Therefore, this study evaluates the whole-plant rape (*B. napus* L.) silage on its quality kinetics, dry matter (DM), and CP degradability, and milk production performance in dairy buffaloes to investigate the changes in forage rape silage during the ensiling process, which can provide a reference for developing and utilizing forage rape silage as a new forage form.

## Materials and Methods

### Forage Rape Culture, Silage Preparation, and Quality Scoring

#### Harvesting and Chopping

Forage rape (*B. napus* L. “Huayouza 62”) from Shahu farm (Xiantao, Hubei, China) sown at a 0.35 kg/mu seed rate and harvested after 196-day growth period was chopped to a length of 50 mm with a chopper machine. The chopped rape was mixed with 7% dry rice straw to adjust the moisture content to 75%, and the chemical analysis is shown in Table 1.

**Table 1:** Fresh forage rape chemical composition [% dry matter (DM)].

DM	16.7
CP	12.05
EE	1.68
CF	28.43
ADF	42.97
NDF	56.19

#### Filling and Sealing

The silage mixture was preserved in the bunker silo, spreading each 20 cm layer to the end of all the filling. After compaction and filling, a layer of polyethylene film is packed on the whole silage bunker, the four corners of the film are inserted between the raw material and the silage pit, and finally, waste tires are used to cover the center, the edges, and the four corners of the canvas to prevent wind blowing.

#### Sampling

Silage samples were taken after 30, 60, and 90 days, samples followed five-point sampling and mixed after each sampling. As opening the silo would cause secondary fermentation, the samples were collected as quickly as possible during sampling and then thoroughly compacted and quickly sealed. After sample collection, the samples were dried and stored at 65°C for standby.

#### Silage Evaluation

The pH of silage samples at different intervals was measured with the pH meter (HI 8424 Shandong Yuesheng Instruments Co., Ltd.), whereas the odor, color, and texture evaluated silage quality. Silage quality parameters were based on the “standard for silage quality evaluation” (Table 2) issued by the Ministry of Agriculture of the People's Republic of China in 1996.

**Table 2:** Silage quality evaluation standards.

**Parameter**	**Total score**	**Excellent**	**Good**	**Generally**	**Poor**
**pH**	25	3.4 (25)	3.9 (17)	4.2 (8)	4.8 and above (0)
		3.5 (23)	4.0 (14)	4.3 (7)	
		3.6 (21)	4.1 (10)	4.4 (5)	
		3.7 (20)		4.5 (4)	
		3.8 (18)		4.6 (3)	
				4.7 (1)	
**Moisture (%)**	20	70 (20)	76 (13)	81 (7)	Above 86 (0)
		71 (19)	77 (12)	82 (6)	
		72 (18)	78 (11)	83 (5)	
		73 (17)	79 (10)	84 (3)	
		74 (16)	80 (8)	85 (1)	
**Color**	20	Turquoise (14–20)	Yellow-green (8–13)	Brownish-yellow (1–7)	Dark brown (0)
**Odor**	25	Sour scent, acceptable (25)	Light sour (9–17)	Pungent wine Sour (1–8)	Moldy (0)
**Texture**	10	Loose and soft Soft and non-sticky (8–10)	Middle (4–7)	Slightly viscosity (1–3)	Sticky (0)
**Total**	100	100–76	75–51	50–26	25 or less
**Grading**					
**Grade**		Excellent	Good	Generally	Poor quality
**Score**		100–76	75–51	50–26	25 or less

### Chemical Analysis

As described previously (12), all samples were dried at 65°C for 48 h to determine the initial moisture content. Then, the samples were ground using a multifunctional pulverizer (Old Bank Boou Hardware Factory, Zhejiang, China) and passed through a 40-mesh sieve (manufactured by Tianxing Wusi Yarn Sieve Factory in Shangyu City, Zhejiang, China) to be stored at 20°C for further analysis. DM, crude ash (CA), CP, ether extract (EE), NDF, and acid detergent fiber (ADF) of the feed were determined according to the Association of Official Analytical Chemists (13), and by the methods described (14, 15) the concentrations of NDF and ADF were determined by the ANKOM Filter Bag Method. In addition, both heat-stable amylase and sodium sulfite were added during NDF extraction (16, 17), and the anthrone sulfate method determined the concentration of water-soluble carbohydrates (WSC) (18).

### Sample Preparation and Incubation

Three fistulated buffaloes (550 ± 20 kg body weight, 4.7 ± 0.76 years) at Shayang buffalo farm, Jingmen, China, were fed two times daily with a total mixed ration (TMR), as shown in Table 3. As described previously (19), the nylon bags were made from nylon fabric (15 cm × 10 cm; 48 μm pore size), sewn with double fine polyester thread to make the bottom of the bag blunt and rounded to prevent the feed samples from being trapped and close the pinhole with the non-dissolvable glue. Next, the bags were heat pressed through an alcohol lamp to guarantee the stability and uniformity of the bag. Next, the nylon rope was inserted into a plastic hose and knotted at both ends to prevent rope breakage. After being labeled by a permanent rumen-stable marker, the nylon bags were filled with 6 g of dry samples, and the weighing of the nylon bags and samples was accurate to 0.0001 g.

**Table 3:** Feed composition and chemical analysis of fistulated buffalo %.

**Feeding composition**	
**Ingredients**	**Content (% of DM)**
Cornstalk silage	30
Peanut vine	10
Rice straw	45
Corn	7
Rice bran	2.5
Soybean meal	3
Wheat bran	2.5
**Chemical composition**	
DM	57.58
OM	89.5
CP	8.95
Ether extract	1.8
CF	26.9
NDF	48.2
ADF	29.9

Next, each silage sample was incubated in three replicates in the rumen, and the incubation time was set for the tested time points. Each sample from the different ensiling times (0, 30, 60, and 90 days) was incubated in the rumen in triplicate for 0, 6, 12, 24, 36, 48, and 72 h; in addition, at each time, the blanks were secured with nylon thread to a piece of string (30 cm long, weight 150 g). After removal, the nylon bags were removed from the mesh bag and placed in a washing machine. The bags were washed repeatedly until the rinse water became clear. Then, the samples were dried in an oven at 65°C until constant weight was reached.

A blank sample is prepared like the previous operations; each sample was packed in three nylon bags but not put into the rumen and treated directly according to the above washing and drying operations.

#### Calculations

The nutrient degradation was calculated as described by the exponential equation (20), which was as follows:


$$\begin{array}{l} D\;=\;a\;+\;b\;\times \;\left(1\;-\;e^{-c\;\times \;t}\right) \end{array}$$

where *D* is the degradation after *t* hours of rumen incubation; *a* is the water-soluble and rapidly degradable fraction; *b* is the insoluble but degradable fraction; and *c* is the degradation rate of the fraction *b*.

The effective degradability (ED) of nutrients was calculated:


$$\begin{array}{l} ED\;=\;a\;+\left(b\;\times \;c\right)\;/\;\left(c\;+\;k\right)\;\left(5\right) \end{array}$$

where *a* is the rapidly degradable fraction (%); *b* is the insoluble but degradable fraction (%); *c* is the degradation rate of the *b* part (%); ED is the effective degradability calculated at a flow rate in the rumen (*K*) 0.06 h^−1^.

### The Dairy Buffalo Feeding Experiment

In this study, 28 crossbred (Mediterranea X Nili-Ravi hybrid) lactating buffaloes (*Bubalus bubalis*; 588 ± 89 kg BW, 6.5 ± 1.3 years) were used to investigate the effects of rape silage inclusion on feed intake, daily milk yield, and milk composition. Animals were randomly assigned to four groups (*n* = 7); each group was randomly divided into four treatment groups (groups C, T1, T2, and T3). Group C was the control group, and rape silage (after 60 days) replaced straw by 10, 20, and 30% (based on DM), respectively, in the diet of TMR groups T1, T2, and T3. The experiment lasted 60 days. The diet concentrate-to-forage ratio was 2:8, supplemented with lick bricks to cover Ca, salt, vitamins, and mineral requirements. The experimental diet content is shown in Table 4. Animals were housed in individual tie stalls and were offered TMR two times daily at 9 a.m. and 3 p.m., for *ad libitum* intake. Animals were fed individually, and feed was weighed and recorded two times a day for each group, and the amount of feed intake was calculated for each group of animals. In addition, milk samples were collected every 20 days from each animal group and stored at 4°C for further determination. Milk samples were mixed 1:1 and conserved with a preservative (0.2 g of bronopol solution/40 ml of milk), kept refrigerated at 4°C, and afterward analyzed for fat, total protein, lactose, and urea at an official milk control laboratory (Hubei Provincial Animal husbandry Bureau, Wuhan, China), using Fourier transform infrared (IR) spectroscopy (MilkoScan 7RM, FOSS Analytical, Hillerød, Denmark) (21).

**Table 4:** Experimental diet content and composition (DM basis).

**Ingredient**	**Groups**			
	**Control**	**10%**	**20%**	**30%**
Corn	5.5	5.5	5.5	5.5
Soybean meal	10	10	10	10
Wheat bran	3.2	3.2	3.2	3.2
Rice straw	65	55	45	35
Brick lick^a^	0.3	0.3	0.3	0.3
Flammulina velutipes	15	15	15	15
Whole-plant rape Silage	0	10	20	30
**Chemical composition (%DM)**				
DM%	74.4	67.2	60.8	54.4
OM	83.2	88.3	88.4	88.6
CP	9.4	11.7	12.5	13.3
EE	1.6	1.7	2.1	2.4
WSC	6.6	16.6	16.2	15.9
CF	25.3	22.0	21.4	20.9
ADF	25.1	22.0	22.2	22.5
NDF	41.6	36.4	36.0	35.6

^a^*A lick brick weighs 5 kg and consists of 98% salt, 25 mg of calcium, 250 mg of phosphorus, 1,000 mg of magnesium, 20 mg of selenium, 150 mg of copper, 50 mg of cobalt, 500 mg of iron, and 200 mg of manganese in each group of four lick bricks*.

### Statistical Analysis

All statistical analyses were analyzed with SAS 9.4 (SAS Institute, Cary, NC, USA, 2017) (22). Data were normally distributed and homoscedastic. Degradation parameters were subjected to the analysis of variance (ANOVA) least significant difference (LSD) procedure. Differences between means were considered significant when *p* < 0.05 for the milk performance study. The following statistical mixed model (repeated measure) was used :


$$\begin{array}{l} Y_{ijk}=\;\mu \;+\;\tau_{i}\;+\;\delta_{ij}\;+\;t_{k}\;+\;{\left(\tau^{*}t\right)}_{ik}\;+\;\varepsilon_{ijk} \end{array}$$

whereas *Y*_*ijk*_ is the dependent variable, μ is the overall mean, τ_*i*_ is the fixed effect of dietary treatment *i, t*_*k*_ is the random effect of time, (τ^*^t)_*ik*_ is the interaction effect of group and time, δ_*ij*_ is the random effect of covariance between repeated measures within an individual, and ε_*ijk*_ is the random error of the variance between the measures within the individual. All reported values are least significant means (LSM), and significance was declared at *p* < 0.05.

## Result

### Effect of Ensiling Duration on Silage Quality

#### Effect of Ensiling Duration on pH

Silage pH at 30, 60, and 90 days were 4.09, 4.08, and 4.39, respectively, and there were no significant differences between ensiling periods. According to the standard of silage quality evaluation, the quality of rape silage ranged from good to average. Silage between 30 and 60 days could keep good quality; however, the quality score dropped after 60 days.

#### The Effects of Ensiling Time on Silage Quality

**Figure 1:**
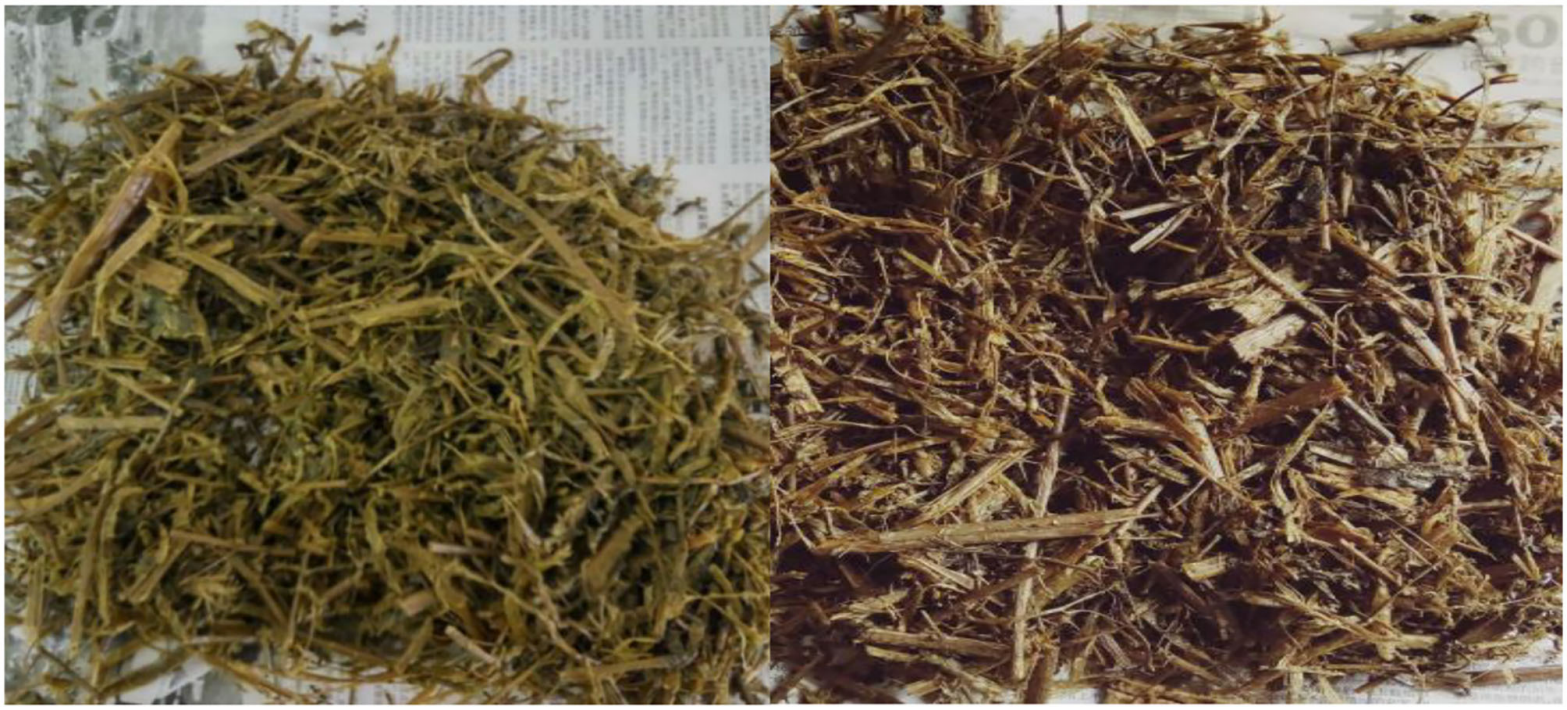
The color of silage rape [**(left)**: silage 60 days; **(right)**: silage 90 days].

Silage quality was comprehensively evaluated by pH, moisture content, and sensory characteristics, including color (Figure 1), odor, and texture. According to the standard of silage quality criteria, we evaluated the effect of ensiling time on silage quality through comprehensive scoring (Table 4). The moisture score increased gradually before 60 days and showed a rapid decrease after day 60. Sensory characteristics scoring results showed that the silage color score was all over 14 before day 60; however, silage odor scores dropped intensely at day 90. Also, the silage was noticed to be sticky at day 90. Finally, the total silage score at days 30 and 60 was 55 and 61, respectively, which achieved a good level (higher than 50). However, silage quality showed a downward trend after day 60 (Table 5), and the score of 31 after 90 days was not a good level, which recommended that the silage needed to be consumed before day 60, which was the optimum quality.

**Table 5:** Effect of ensiling duration on the comprehensive sensory investigation score.

**Ensiling time**	**pH**	**Moisture**	**Color**		**Smell**		**Texture**		**Comprehensive score**	
	**Score** **(Mean)**	**Score** **(Mean %)**	**Color**	**Score**	**Smell**	**Score**	**Texture**	**Score**	**Level**	**Total** **score**
30 days	10 (4.1)	7 (81.2)	Green	14	Weak aromatic	16	Loose & soft	8	Good	55
60 days	10 (4.1)	13 (76.91)	Green	15	Weak aromatic	16	Medium	7	Good	61
90 days	5 (4.4)	6 (82.21)	Yellow green	9	Pungent	7	sticky	4	low	31
SEM	0.55	1.15	Different letters indicated a significant difference was indicated by (*p* <0.05),							
*p*-Value	>0.05	>0.05	while the remaining indicated no significant difference (*p* > 0.05)							

#### The Influence of Ensiling Time on Nutrient Composition

Silage samples were collected from different ensiling timepoints for proximate chemical analysis (Table 6). The CP content of silage showed an upward trend from 0 to 30 days and a downward trend from 60 to 90 days. On the other hand, the EE content is relatively low from 0 to 30 days but increases from 60 to 90 days; it was the highest after 60 days (4.33%). Although the average EE content increased 1.7 times (*p* < 0.05) compared with fresh rape (day 0), there was a decline in NDF among treatments after ensiling (*p* < 0.05), and it reached its lowest value between 30 and 60 days (46.75 and 47.82%, respectively). Also, ADF decreased significantly (*p* < 0.05) during ensiling; it reached its lowest value between 30 and 60 days (30.70 and 34.14%, respectively). Thus, NDF and ADF reached their lowest level at the early ensiling period (30–60 days) and gradually increased as fermentation time was prolonged.

**Table 6:** Effect of ensiling duration on the rape silage nutrient content.

**Nutrient component**	**Control (Day 0)**	**Fermentation time**			**SE*M***	***p*** **-Value**		
		**30 days**	**60 days**	**90 days**		**Diet**	***L***	***Q***
DM	19.45	17.89	21.32	17.48	0.14	0.45	0.71	0.32
CP	12.05	12.32	12.28	12.00	0.11	0.71	0.86	0.27
EE	2.18^c^	2.77^b^	4.33^a^	4.15^a^	0.27	<0.001	<0.001	<0.001
NDF	56.19^a^	46.57^c^	47.82^c^	52.62^b^	1.25	0.001	0.08	<0.001
ADF	42.97^a^	30.70^c^	34.14^bc^	37.14^b^	1.44	<0.001	0.02	<0.001

*CP, crude protein; EE, ether extract; CF, crude fiber; ADF, acid detergent fiber; NDF, neutral detergent fiber*.

*Differences between treatment and period were considered significant at p < 0.05*.

### Effect of Ensiling Duration on the DM and CP *in situ* Degradability

These data from the *in situ* degradability results for DM and CP from different ensiling time points can reflect nutrient intake in animals.

With the prolongation of silage time, compared with 0 (no change), the instantaneous DM degradation rate decreased significantly at 0, 6, 12, 24, 36, 48, 60, and 72 h. The instantaneous degradation of the rumen degradation rate of DM for 0–24 h was 0 > 30 > 90 > 60 days, and that for 36–72 h was 0 > 30 > 60 > 90 days (Tables 7,8). However, the instantaneous degradation of the rumen degradation rate of CP showed no significant differences.

**Table 7:** Effect of the ensiling duration on average DM degradability of forage rape silage after 0, 6, 12, 24, 36, 48, 60, and 72 h incubation *in situ* technique.

**Incubation time (h)**								
**Ensiling days**	**0**	**6**	**12**	**24**	**36**	**48**	**60**	**72**
0	27.082^a^	44.726^a^	39.6^a^	60.12^a^	72.6^a^	80.4^a^	85.074^a^	82.12^a^
30	24.62^a^	40.66^b^	36^ab^	54.66^b^	66.41^b^	70.34^ab^	77.34^b^	74.66^b^
60	13.413^b^	23.8^c^	30.6^b^	42.5^c^	52.7^c^	60.63^b^	65.739^bc^	70.26^b^
90	13.96^b^	28.38^c^	38.93^a^	46.66^c^	48.8^c^	53.06^c^	60.26^c^	65.06^c^
S.E.M	5.66	7.25	7.59	6.85	8.46	8.65	9.95	5.6
*p*-Value	<0.05							

*Means within the same column with different superscripts differ (p <0.05)*.

**Table 8:** Effect of ensiling duration on average crude protein (CP) degradability of forage rape silage after 0-, 6-, 12-, 24-, 36-, 48-, 60-, and 72-h incubation *in situ* technique.

**Incubation time (h)**								
**Ensiling days**	**0**	**6**	**12**	**24**	**36**	**48**	**60**	**72**
0	25.41	31.67	39.00	46.00	48.33	50.33	56.67	58.33
30	26.57	37.00	46.67	48.67	49.67	48.67	56.00	55.33
60	20.32	28.33	36.33	40.00	47.33	48.33	49.67	44.67
90	22.13	38.67	40.00	44.33	52.33	51.33	52.67	59.00
S.E.M	1.45	2.39	2.20	1.82	1.08	0.71	1.62	1.45
p-Value	>0.05							

*Different letters indicated a significant difference (p < 0.05), while the remaining indicated no significant difference (p > 0.05)*.

As, at day 90 of ensiling, DM ED was significantly dropped (*p* < 0.05), the degradation rate of silage DM at both 90 and 60 days was significantly lower than (*p* < 0.05) after 30 days of silage. The DM effective degradation rate showed a decreasing trend from 0 to 90 days after ensiling; and a significant decrease after 90 days. However, the CP effective degradation rate showed no significant differences from different ensiling time samples (*p* > 0.05) (Tables 9,10).

**Table 9:** Effect of ensiling duration on the rapidly degradable nutrient (a, %), slowly degradable nutrient (b, %), effective degradation rate (c), and the effective degradability (ED, %) of forage rape silage.

**Ensiling days**	**a**	**b**	**c**	**ED**
0	15.53^c^	36.45^c^	0.0493^a^	39.72^b^
30	23.37^b^	47.62^b^	0.0427^b^	35.83^b^
60	27.8^a^	58.44^a^	0.0363^c^	43.33^a^
90	15.41^c^	54.89^b^	0.035^c^	24.30^c^
SEM	1.38	1.85	0.003	1.30
*p*-Value	0.04	0.02	0.0234	0.009

*The different letters means significant difference (P < 0.05)*.

**Table 10:** Effect of ensiling duration on the rapidly degradable nutrient (a, %), slowly degradable nutrient (b, %), effective degradation rate (c), and the ED (%).

**Ensiling days**	**a**	**b**	**c**	**ED**
0	56.70	41.47	0.055	41.56
30	60.47	37.34	0.0290	42.86
60	62.13	36.69	0.0363	44.33
90	54.64	42.45	0.0297	41.56
SEM	1.52	1.03	0.008	1.13
*p*-Value	0.359	0.050	0.393921	0.384

*Different letters indicated a significant difference (p < 0.05), while the remaining indicated no significant difference (p > 0.05)*.

### Effect of Silage Inclusion on the DM Intake, Milk Yield, and Composition

Rape silage was included in the TMR at 10%, 20%, and 30% of the total DM. Although DM intake (DMI) and concentrations of milk lactose and urea nitrogen were not affected by treatment (*p* > 0.05), fat and protein content increased linearly (*p* < 0.05) (Table 11).

**Table 11:** Effects of rape silage inclusion on dry matter intake (DMI) and milk composition of lactating buffaloes.

**Items**	**Treatment**				**SEM**	***p*** **-Value**		
	**Control**	**10%**	**20%**	**30%**		**Diet**	***L***	***Q***
Dry matter intake, DMI (kg)	21.12	19.93	19.45	19.92	3.38	0.81	0.47	0.52
Milk yield (kg/day)	3.39	3.82	3.20	3.38	0.48	0.98	0.89	0.91
Milk fat, %	7.39 ^b^	7.83 ^b^	8.21 ^a^	8.22^a^	0.14	0.14	0.03	0.43
Milk protein, %	4.60^c^	5.15^b^	5.16^b^	5.35^a^	0.11	0.11	0.03	0.40
Milk lactose, %	5.49	5.46	5.47	5.32	0.06	0.79	0.40	0.65
Urea nitrogen, mg/dl	11.15	12.20	13.35	12.60	0.37	0.27	0.16	0.22

*The different letters means significant difference (P < 0.05)*.

## Discussion

### Effects of Ensiling Time on Silage Sensory Characteristics

There are many microbes on the silage surface; some are beneficial to silage, e.g., lactic acid bacteria, but others are more harmful, e.g., spoilage bacteria, yeasts, and molds. To obtain optimum silage quality, the amount of lactic acid bacteria in the silage material needs to reach 5 log to 6 log colony forming units/g (CFU/g) of fresh weight (23, 24). In addition, bacterial lactic acid maintains the silage system in a stable pH range, which is closely related to silage fermentation activity, thus reducing the pH value and increasing silage quality. Furthermore, the optimum pH for the protease activity in silage is around 6, and the enzyme activity decreases when pH is 4–6, the protease activity at pH 4 is 15%−35% at pH 6 (25). Therefore, the pH value and its rate of decline during silage will determine the final silage quality. In corn silage, the silage quality grade is excellent, good, average, and low when the pH is between 3.8 and 4.2, between 4.1 and 4.3, between 4.4 and 5.0, and higher than 5.0, respectively (26).

During ensiling, the silage pH ranged between 4 and 4.3, and the pH value of whole-plant rape silage in 30–60 days is lower in the whole silage period. This indicates that direct silage in that period, whole-plant rape, can maintain a moderate amount of beneficial bacterial colonies and achieve a good acidity balance. Moreover, we scored the five indicators of moisture, pH, color, odor, and texture of silage, referring to the “silage quality assessment standard.” During the 30–60 days ensiling, the silage color is green, similar to the color of raw materials; the odor is sour, the texture is loose, smooth, and non-sticky; the total score reached the maximum in 60 days. However, the total score drops rapidly after 60 days and has a sticky texture and pungent odor at 90 days. Therefore, rape silage could maintain good quality between 30 and 60 days of ensiling, and it is recommended to open the cellar at 60 days; silage quality should be optimized.

### Effect of Ensiling on Silage Kinetics and Ruminal Degradability

During the ensiling process, the fermentation activity of lactic acid bacteria prevented CP loss, and could effectively maintain the CP content of rape. Also, the EE content was significantly higher at 60–90 days than at 0–30 days, and in the control group, the silage EE content at 60 and 90 days increased by 2.65 and 2.47, respectively, which was 1.5 times higher than the control after ensiling 60 days. Moreover, the ensiling process increased protoplasm osmosis with the death of plant cells; therefore, the nutrient concentration effect of soluble nutrients with water loss might increase EE concentration. On the other hand, it was noticed that both NDF and ADF of silage decreased significantly after ensiling 30–60 days. While NDF is an essential indicator for fiber quality and for determining the appropriate diet ratio, ADF is the crucial indicator of forage energy; its low content can facilitate feed digestion. Also, NDF digestibility is slow, which is generally considered a significant factor affecting DMI (27, 28). Although NDF content is negatively correlated with non-fiber carbohydrates (NFC) (29), plant carbohydrates are either oligosaccharides or their polymers, such as cellulose and starch, are the primary substrates for fermentation by rumen microbial activity (30). Therefore, the higher concentration of WSC might also contribute to a higher degradability of DM and organic matter (OM) in silage (31). The latter factors may explain the rapid decline in NDF and ADF due to some microbial cellulase activity (26); lactobacillus activity in silage fermentation consumed a certain amount of WSC, making the ratio of NDF and ADF to increase relatively slowly afterward.

Although nutrient changes do not necessarily reflect the nutritional value of silage, they can give an idea about the extent of intake and utilization; therefore, a rumen nylon bag is the most effective method to evaluate the degradation rate in the *in vivo* environment (20, 32). The concentration of cell wall components in forages is highly variable according to the variety and stage of plant maturity; lignin concentration increases with plant maturation and reduces cell wall degradability (33). NDF degradability is limited primarily by the cross-linking of lignin with other fibrous components (33). In theory, cellulose and hemicellulose can be completely digested by ruminants, but the ester bond formed by lignin and hemicellulose encapsulates cellulose (34). Thus, lignin cannot be entirely degraded by rumen microorganisms, which affects the digestion and utilization of cellulose and hemicellulose. Presumably, the ensiling process increased the cross-linking of lignin in rape silage with other fiber compounds (35), resulting in reduced microbial access and, therefore, less crude fiber degradability and, in turn, DM degradability.

### Effect of Silage Inclusion on the Milk Production Response

One of the most valuable advantages of buffaloes is their increased ability to utilize unpalatable agricultural by-products, which can be further incorporated into rations because their coarser texture, odors, and flavors are ameliorated by ensiling (11, 36, 37). In this study, we included rape silage in the dairy buffalo diet; the difference in DMI was not significant in each group, which showed that the whole-plant rape silage did not reduce the diet's palatability. NDF is an essential factor limiting DMI; when NDF is more than 35%, it may inhibit DMI by physical rumen filling; when it is lower than 25%, energy intake becomes the main factor limiting DMI (38, 39).

On the other hand, milk fat and protein rates increased linearly with whole-plant rape silage (*p* < 0.05), reflecting the feeding value of rape silage for dairy buffaloes. There is a positive correlation between a linear increase in EE content, degradability of rape silage, and a significant increase in milk fat percentage. Also, the milk protein rate increased linearly (*p* < 0.05) as the proportion of addition increased; these results could be supported by non-significant changes in degradation rate and palatability after ensiling.

Moreover, the difference in milk lactose and milk urea nitrogen (MUN) is not significant; it may be linked to a decrease in DM degradability. However, the MUN content of each treatment group was higher than that of the control group. It has been reported that 87% of the (MUN) variation is caused by nutritional factors and 13% by non-nutritive factors (40); MUN is essential for assessing dietary protein and energy levels (41, 42). MUN concentration is correlated with CP, rumen degradable protein (RDP), undegradable protein (UDP), non-fiber carbohydrates (NFC), and energy protein ratios in the diet (43–45). The normal range of MUN concentration in milk is 10–15 mg/dl (46, 47); the urea nitrogen level of the whole-plant rape silage treatment group in this study was within this range. Although the difference in MUN in each group was not significant, the replacement of whole-plant rape silage had no effect on buffalo's rumen N energy balance. However, the sample needs to be expanded further to confirm this trend.

## Conclusions

Whole-plant rape ensiling can effectively maintain CP, increase EE content, and reduce NDF and ADF content. Silage reaches optimum silage quality between 30 and 60 days of ensiling; thus, the right time to open the cellar can be set in this period. Whole-plant rape silage significantly reduces DM degradability, but does not change CP degradability. In addition, whole-plant rape silage linearly increased milk fat content, protein level, lactose level, and MUN did not change significantly after ensiling rape. Therefore, whole-plant rape silage can be used as a good forage source; however, improvements of its species and ruminal microbial interactions with fatty acid changes need to be further examined.

## Data Availability Statement

The original contributions presented in the study are included in the article/supplementary material, further inquiries can be directed to the corresponding authors.

## Ethics Statement

The animal study was reviewed and approved by the Ethical Committee of the Hubei Research Center of Experimental Animals [Approval ID: SCXK (Hubei) 20080005].

## Author Contributions

Conceptualization, project administration, funding acquisition, and supervision: YL and HG. Methodology, writing-review, and editing: MA, ZD, YL, and HG. Formal analysis: WW, ZD, and MA. Investigation: WW, AS, ZD, MA, HL, WL, ZA, ZG, and WB. Writing-original draft preparation: ZD and MA. All authors have read and agreed to the published version of the manuscript.

## Funding

This study was supported by National Natural Science Foundation of China (No. 32172731), Fundamental Research Funds for the Central Universities (No. 2662022DKYJ002) the earmarked fund for CARS36, and Hubei Key Research and Development Program (No. 2020BC001).

## Conflict of Interest

The authors declare that the research was conducted in the absence of any commercial or financial relationships that could be construed as a potential conflict of interest.

## Publisher's Note

All claims expressed in this article are solely those of the authors and do not necessarily represent those of their affiliated organizations, or those of the publisher, the editors and the reviewers. Any product that may be evaluated in this article, or claim that may be made by its manufacturer, is not guaranteed or endorsed by the publisher.
